# Effects of a Differential Diagnosis List of Artificial Intelligence on Differential Diagnoses by Physicians: An Exploratory Analysis of Data from a Randomized Controlled Study

**DOI:** 10.3390/ijerph18115562

**Published:** 2021-05-23

**Authors:** Yukinori Harada, Shinichi Katsukura, Ren Kawamura, Taro Shimizu

**Affiliations:** 1 Department of General Internal Medicine, Nagano Chuo Hospital, Nagano 380-0814, Japan; yharada@dokkyomed.ac.jp; 2 Department of Diagnostic and Generalist Medicine, Dokkyo Medical University, Tochigi 321-0293, Japan; katukura@dokkyomed.ac.jp (S.K.); renkawa@dokkyomed.ac.jp (R.K.)

**Keywords:** artificial intelligence, automated medical-history-taking system, commission errors, diagnostic accuracy, differential-diagnosis list, omission errors

## Abstract

A diagnostic decision support system (DDSS) is expected to reduce diagnostic errors. However, its effect on physicians’ diagnostic decisions remains unclear. Our study aimed to assess the prevalence of diagnoses from artificial intelligence (AI) in physicians’ differential diagnoses when using AI-driven DDSS that generates a differential diagnosis from the information entered by the patient before the clinical encounter on physicians’ differential diagnoses. In this randomized controlled study, an exploratory analysis was performed. Twenty-two physicians were required to generate up to three differential diagnoses per case by reading 16 clinical vignettes. The participants were divided into two groups, an intervention group, and a control group, with and without a differential diagnosis list of AI, respectively. The prevalence of physician diagnosis identical with the differential diagnosis of AI (primary outcome) was significantly higher in the intervention group than in the control group (70.2% vs. 55.1%, *p* < 0.001). The primary outcome was significantly >10% higher in the intervention group than in the control group, except for attending physicians, and physicians who did not trust AI. This study suggests that at least 15% of physicians’ differential diagnoses were affected by the differential diagnosis list in the AI-driven DDSS.

## 1. Introduction

Diagnostic errors are challenging problems to solve in clinical practice [1]. For reducing diagnostic errors, interventions to multiple factors and steps in the diagnostic process are warranted. Improving the quality of differential diagnosis can play a major role in the better diagnostic process. Differential diagnosis is the cognitive process of producing and prioritizing a list of potential diagnoses for a given clinical presentation [2]. By conducting efficient differential diagnosis at the early phase of the diagnostic process, physicians can reach the correct diagnosis timely. For example, when a 50-year-old man presents to the emergency department with acute onset severe chest pain, a physician may consider acute coronary syndrome as a most likely diagnosis. At the same time, if the physician may also consider acute aortic dissection and pulmonary thromboembolism as other potential diagnoses, the physician can reconstitute a diagnostic plan quickly when the patient does not have acute coronary syndrome.

A diagnostic decision support system (DDSS) that supports the diagnostic process by generating differential diagnoses from observations provided [3] is expected to reduce diagnostic errors by reducing cognitive biases and reinforcing physicians’ knowledge. It is said that generating potential diagnoses is a rate-limiting step of differential diagnosis for novice diagnosticians [2], and their diagnostic accuracy was reported to be associated with the number of generated diagnoses [4]. Besides, using a simple differential diagnosis checklist, which could remind users of some missed diagnoses, was reported to improve the diagnostic accuracy of medical students [5]. Therefore, the DDSS, which can generate effective differential diagnoses respective to each clinical presentation, seems a promising technology for improving diagnosis. Furthermore, the DDSS has a strength that can filter and prioritize the differential diagnosis, similar to the diagnostic process of experienced diagnosticians. Previous studies reported that the DDSS has high accuracy [6,7,8] and improved the diagnostic accuracy of physicians or medical students [8,9].

The DDSS, however, also has several limitations. The most important limitation seems the fact that even when the correct diagnosis is listed in the differential diagnosis list of the DDSS, the correct diagnosis cannot always be placed on the top. Physicians usually consider around 3 (depends on the situation) diagnoses as actually to be investigated in the daily clinical practice. Therefore, assuming the DDSS, which provides the top 20 differential diagnoses, physicians may only choose around three diagnoses from the list for actual differential diagnoses. As a result, physicians might not be able to choose the correct diagnosis from the differential diagnosis list of the DDSS. Besides, because there is a possibility that the DDSS fails to generate the correct diagnosis, physicians should also generate the differential diagnoses which are not included in the list of the DDSS. However, physicians sometimes skip the step, which results in missing the correct diagnosis.

As noted above, diagnostic errors can occur even with the use of a DDSS. Some errors specifically related to DDSSs, such as “omission errors,” which occur when physicians do not accept the correct suggestion from the DDSS, and “commission errors,” which occur when physicians accept the incorrect suggestion from the DDSS, should be addressed to improve the use of DDSSs [10]. The frequencies of omission and commission errors have been studied for a type of DDSS that generated differential diagnoses after physicians enter the key symptoms and findings after a clinical encounter [8]. In this type of DDSS, the initial diagnosis by a physician before DDSS consultation may cause a bias in that physicians only accept the suggestion from the DDSS when this is consistent with the idea of the physician, resulting in omission and commission errors [11]. In addition, the initial diagnosis was reported to be important for accurate diagnosis [12,13], and the accuracy was high in physicians who received enjoy early support with a DDSS [14,15,16]. Therefore, the other types of DDSS that generate differential diagnosis from information entered by patients themselves before clinical encounter [10,17] can be expected to reduce DDSS-related diagnostic errors, particularly omission errors.

Despite this hypothesis, a recent study suggested some concerns about DDSS-related diagnostic errors even when using such a new type of DDSS. In that study, an artificial intelligence (AI)-driven DDSS with an AI-driven automated medical history-taking (AMHT) system was used. This AI-driven DDSS with an AI-driven AMHT system provides differential diagnosis lists with a summary of the clinical history generated from patient-entered information. As a result, that study showed that the AI-driven DDSS with AI-driven AMHT system also led to omission and commission errors at similar rates as did the type of DDSS that generated differential diagnoses after physicians enter the key symptoms and findings after a clinical encounter [10]. The following study, which we reported recently, also confirmed the findings [17]. However, these studies did not assess the mechanism of omission and commission errors when using this new type of DDSS. For example, regarding the fact that omission errors were not decreased in this new type of DDSS, there were at least two possible reasons. First, contrary to the expectation, physicians did not accept the diagnoses of the DDSS at a high rate. Second, although physicians accepted the diagnoses of the DDSS at a high rate, physicians could not choose the correct diagnosis from the differential diagnosis list of the DDSS. Therefore, the prevalence of diagnoses from the DDSS in physician diagnoses when using this new type of DDSS should be clarified.

In this study, we aimed to assess the prevalence of diagnoses from AI-driven DDSS in physicians’ differential diagnoses when using DDSS, which generates differential diagnoses from the information entered by the patient before the clinical encounter, by analyzing the differential diagnoses made by physicians who participated in a previously conducted randomized controlled study.

## 2. Materials and Methods

### 2.1. Study Design, Participants, Materials, and Intervention

The primary analysis results of our randomized controlled study were previously reported [17]. In brief, this randomized controlled study was conducted in January 2021 at the Department of Diagnostic and Generalist Medicine of Dokkyo Medical University, in which 22 physicians (five interns, eight residents, and nine attending physicians) were randomly assigned in a 1:1 allocation to either reading 16 written clinical vignettes generated by AI on the basis of the information of actual patients with (intervention group, *n* = 11) or without (control group, *n* = 11) a top-10 differential diagnosis list of AI. The AI was trained to suggest differential diagnoses based on supervised machine learning. The clinical vignettes used in the study only included patient history and vital signs. The confirmed diagnoses of 16 vignettes were asthma, diverticulitis, heart failure (2 cases), hyperosmolar hyperglycemic state, ischemic colitis, myocarditis, pancreatitis (2 cases), peritonsillar abscess, pneumonia (2 cases), pneumothorax, pyelonephritis, subacute myocardial infarction, and type 2 diabetes mellitus. The correct diagnosis was listed in the top-10 differential diagnosis list of AI in eight clinical vignettes (AI correct cases). The correct diagnosis was not listed in the top-10 differential diagnosis list of AI in the other eight clinical vignettes (AI incorrect cases). The participants were required to write up on the answer sheet up to three likely differential diagnoses, from the most to the least likely, for each vignette within two minutes by reading the vignettes. The study was approved by the bioethics committee of Dokkyo Medical University (2020-018) and the research ethics committee of Nagano Chuo Hospital (NCH20-11). The study was registered with UMIN-CTR (trial registration No. UMIN000042881). Written consent was obtained from all the participants.

### 2.2. Data Collection and Outcomes

Data were collected on the participants’ sex, experience (intern, resident, or attending physician), trust in the AI-driven DDSS (Yes or No), and participants’ diagnoses.

The primary outcome of this exploratory analysis was the prevalence of physician answers identical to the diagnoses in the differential diagnosis lists of AI. Two reviewers (YH and RK) independently classified all the diagnoses provided for each vignette as either identical or not to the diagnosis in the differential diagnosis list of AI. Discordant classifications were resolved by the third reviewer (SK).

### 2.3. Statistical Methods

Continuous data are presented as median with 25th and 75th percentiles. Categorical data are presented as counts and proportions (%). Baseline characteristics and the primary outcome between the intervention and control groups were compared using the chi-square test. All the *p*-values in the statistical tests were two-tailed, and *p*-values < 0.05 were considered statistically significant. All the statistical analyses were performed using R version 3.6.3 (The R Foundation for Statistical Computing, Vienna, Austria).

## 3. Results

### 3.1. Baseline Characteristics of Physicians Who Participated in the Study

Of the physicians in the intervention and control groups, 7 (63.6%) and 9 (81.8%) were male, respectively (*p* = 0.41). Experience of physicians was not different between the two groups (*p* > 0.99): 3 (27.2%) in the intervention group and 2 (18.2%) in the control group were interns; 4 (36.4%) in the intervention group and 4 (36.4%) in the control group were residents, and 4 (36.4%) in the intervention group and 5 (45.4%) in the control group were attending physicians. Six physicians (54.5%) in the intervention group and seven physicians (63.6%) in the control group responded that they trusted the AI-driven DDSS (*p* = 0.70).

### 3.2. The Number of Physician Diagnoses

Physicians generated 975 diagnoses through the study: 490 from the intervention group and 485 from the control group. If all physicians had generated three diagnoses in every vignette, a total amount of diagnosis would have been 1056 (22 physicians × 16 vignettes × 3 diagnoses). Therefore, the total fill rate of answer boxes in the study, calculated as the actual number of physician diagnoses divided by the possible maximal number of physician diagnoses, was 92.3% (975/1056). As shown in Table 1, the fill rates were not different between the intervention and control groups in any subgroups except for intern (93.8% in the intervention group vs. 83.3% in the control group, *p* = 0.02). In the intervention group, the fill rates were not different between male and female (*p* = 0.81); among interns, residents, and attending physicians (*p* = 0.73); between physicians who trusted AI or not (*p* = 0.94); and between AI-correct and AI-incorrect cases (*p* = 0.87). In the control group, the fill rates among interns, residents, and attending physicians were significantly different (*p* < 0.001); while there was no difference between male and female (*p* = 0.13); between physicians who trusted AI or not (*p* = 0.54); and between AI-correct and AI-incorrect cases (*p* = 0.11).

### 3.3. Primary Outcome

The inter-rater agreement between the two independent evaluators was 0.98 (kappa coefficient) for whether the answers were identical to the diagnoses of AI. The prevalence of physician diagnosis identical with the differential diagnosis of AI was significantly higher in the intervention group (344/490, 70.2%) than in the control group (267/485, 55.1%), and the absolute difference was 15.2% (95% confidence interval, 8.9–21.4%; *p* < 0.001). This result was consistent when limited to the most, second, and third likely diagnoses in the physicians’ differential diagnosis list and when limited to the subsets of AI correct and incorrect cases (Table 2). The absolute difference in physician diagnosis identical with the differential diagnosis of AI between the intervention and control groups was largest in rank 3 (rank 1, 10.2%; rank 2, 17.6%; and rank 3, 18.6%). In the intervention group, while the prevalence of physician diagnosis identical with the differential diagnosis of AI was around 30% lower in the AI-incorrect cases than the AI-correct cases, even 56% of physician diagnosis was identical with the wrong differential diagnosis in the AI-incorrect cases.

### 3.4. Subgroup Analysis

In the subgroup analysis, the prevalence of physician diagnosis identical with differential diagnosis of AI was significantly higher in the intervention group than in the control group except for the females (although the difference between the groups was 12.8%), attending physicians, and those who did not trust AI (Table 3). The prevalence of physician diagnosis identical with differential diagnosis of AI was numerically higher in male than female, higher in intern than resident and attending physician, and higher in physicians who trusted AI than who did not trust AI, while these numeric differences were not observed in the control group.

## 4. Discussion

The present study revealed that the prevalence of diagnoses identical with the differential diagnosis of AI was significantly higher in the physicians who read clinical vignettes with differential diagnosis lists of AI than in those who read clinical vignettes without a differential diagnosis list of AI. The difference in the prevalence of physician diagnosis identical with the differential diagnosis of AI between the two groups was not statistically significant in the subgroups of female, attending physicians, and physicians who did not trust AI.

By utilizing data from a randomized controlled trial, this study clarified the actual effect of a type of DDSS that generates a differential diagnosis from the information entered by the patient before a clinical encounter on the physician’s differential diagnosis. In this study, while the total prevalence of diagnosis identical with the differential diagnosis of AI in the intervention group was 70%, considering that 55% of the differential diagnoses of the physicians who had no access to a differential diagnosis list of AI were also identical with the differential diagnoses of AI, the DDSS seemed to have affected the physicians’ diagnoses by at least 15%.

The difference of 15% between the total prevalence of diagnosis identical with the differential diagnosis of AI in the two groups was consistent with those reported in the prior studies. Previous studies showed that approximately 15% of physicians’ differential diagnoses changed between before and after DDS consultation [11,18,19]. These results suggest that regardless of using the DDSS before or after the patient encounter, approximately 15% of the physicians’ differential diagnoses can be affected by the differential diagnosis list generated by the DDSS. According to a previous study, task performance can worsen by using an automated rather than a manual procedure when the reliability of automation is <70% [20]. As the reliability of AI in this study was set at 50% (the correct diagnosis was listed in 8 of 16 cases), approximately 15% of the effect of the DDSS on the physicians’ differential diagnosis may be associated with the commission errors observed in our main analysis [17]. The prevalence of diagnoses identical with the differential diagnosis of AI was also approximately 15% higher in the intervention group than in the control group, even in the subset of AI incorrect cases, which led to commission errors.

In the two subgroups that included the attending physician and physician who did not trust AI, a difference in the prevalence of diagnosis identical with the AI-driven differential diagnosis of only <10%, which was not significant, was observed between the intervention and control groups. These results were consistent with previous reports that physicians who were more experienced and had less trust in automation were reluctant to accept the advice from automated decision support systems [21,22,23]. These two groups were associated with fewer commission errors in our main analysis [17]; therefore, to reduce commission errors when using a DDSS, physicians should consider the reliability of DDSSs, and less experienced physicians or physicians who trust AI should reduce their reliance on AI.

In the study, there was around a 30% difference in the prevalence of diagnoses identical with the differential diagnosis of AI between AI-correct and AI-incorrect cases in the intervention group. This means that physicians could discriminate whether the correct diagnosis was in the list of AI or not to somewhat degree. Some participants in the study said, “In some cases, I felt that the list by AI was wrong this was from my intuition, though.” However, as many as 56% of physician diagnoses were identical with the AI diagnoses in the AI-incorrect cases, the physicians’ discrimination skill was too far from ideal. Indeed, some participants noted, “Some of my diagnoses might have been affected by the AI list when I could not judge whether AI was correct or not.” To enhance the physicians’ skill in discriminating whether the AI is correct or not, one possible solution may be to visualize the process and evidence in the generation of AI differential diagnoses (opening the black box of AI), which is called explainable AI [24,25]. Explainable AI refers to, in a nutshell, AI in which the output by the AI can be logically understood and explained by humans. Therefore, if physicians can see that an AI diagnosis comes from only nonspecific symptoms and not from cardinal symptoms, physicians may avoid choosing the diagnosis.

Interestingly, most of the residents and attending physicians who participated in the study said that they generated differential diagnoses themselves and then checked the list of AI. In contrast, most interns said they checked the AI list soon after reading the vignette and generated differential diagnoses. Therefore, although the DDSS that generates differential diagnoses from information entered by patients themselves before the clinical encounter is expected to reduce the bias from the initial physician diagnosis, that seemed not to work in residents and attending physicians in the study [17]. Enhancing the explainability of AI may be a solution to this problem. High explainability of AI can increase the trust of physicians for AI, and it may change the process in the generation of differential diagnosis in physicians when using the AI-driven DDSS.

The present study has several limitations. First, it was an exploratory analysis of data from a randomized controlled study, and the primary outcome was not prespecified. Although the fill rates were not statistically different between the intervention and control groups except for intern, the actual number of diagnoses generated by physicians was widely different in some subgroups between the intervention and control groups. In particular, the actual number of diagnoses generated by physicians in females and interns in the control group seems relatively low. These facts may have biased the outcomes, especially in subgroup analyses. Some subgroup analyses might have been statistically underpowered (e.g., the inclusion of females in the subgroup analysis). Second, the study was not conducted in a real clinical practice setting. Third, the participants were only general physicians and interns from a single institution. Therefore, our study results should be validated in other populations.

## 5. Conclusions

The prevalence of diagnoses identical with the differential diagnosis of AI was significantly higher in the physicians who read clinical vignettes with differential diagnosis lists of AI than in those who read clinical vignettes without a differential diagnosis list of AI. Attending physicians and physicians who did not trust AI were reluctant to accept the differential diagnosis of AI.

## Figures and Tables

**Table 1:** The fill rates of answer boxes with physician diagnoses.

	With AI Differential List	Without AI Differential List	*p* Value
Total	490/528 (92.8%)	485/528 (91.9%)	0.64
The rank of physician diagnosis			
1	176/176 (100%)	176/176 (100%)	>0.99
2	173/176 (98.3%)	172/176 (97.7%)	>0.99
3	141/176 (80.1%)	137/176 (77.8%)	0.69
Sex			
Male	313/336 (93.2%)	401/432 (92.8%)	0.97
Female	177/192 (92.2%)	84/96 (87.5%)	0.28
Experience			
Intern	135/144 (93.8%)	80/96 (83.3%)	0.02
Resident	176/192 (91.7%)	185/192 (96.4%)	0.09
Attending physician	179/192 (93.2%)	220/240 (91.7%)	0.67
Trust in AI			
Yes	268/288 (93.1%)	311/336 (92.6%)	0.93
No	222/240 (92.5%)	174/192 (90.6%)	0.60
AI correctness			
AI correct	246/264 (93.2%)	237/264 (89.8%)	0.21
AI incorrect	244/264 (92.4%)	248/264 (93.9%)	0.60

data are presented as the actual number of physician diagnoses/possible maximal number of physician diagnoses.

**Table 2:** Prevalence of physician diagnosis identical with the differential diagnosis of AI.

	With AI Differential List	Without AI Differential List	*p* Value
The rank of physician diagnosis			
1	141/176 (80.1%)	123/176 (69.9%)	0.04
2	116/173 (67.1%)	85/172 (49.4%)	0.001
3	87/141 (61.7%)	59/137 (43.1%)	0.003
AI correctness			
AI correct	207/246 (84.1%)	168/237 (70.9%)	<0.001
AI incorrect	137/244 (56.1%)	99/248 (39.9%)	<0.001

data are presented as the number of physician diagnoses identical with the differential diagnosis of AI/the total number of physician diagnoses.

**Table 3:** Prevalence of physician diagnosis identical with the differential diagnosis of AI in the subgroups.

	With AI Differential List	Without AI Differential List	*p* Value
Sex			
Male	230/313 (73.5%)	224/401 (55.9%)	<0.001
Female	114/177 (64.4%)	43/84 (51.2%)	0.06
Experience			
Intern	107/135 (79.3%)	42/80 (52.5%)	<0.001
Resident	123/176 (69.9%)	101/185 (54.6%)	0.004
Attending physician	114/179 (63.7%)	124/220 (56.4%)	0.17
Trust in AI			
Yes	209/268 (78.0%)	171/311 (55.0%)	<0.001
No	135/222 (60.8%)	96/174 (55.2%)	0.30

data are presented as the number of physician diagnoses identical with the differential diagnosis of AI/the total number of physician diagnoses.

## Data Availability

The data presented in this study are available on request from the corresponding author. The data are not publicly available due to privacy restrictions.
